# Predators Show Seasonal Predilections for Model Clay Spiders in an Urban Environment

**DOI:** 10.1038/s41598-018-30778-y

**Published:** 2018-08-20

**Authors:** L. D. Mason, G. Wardell-Johnson, S. J. Luxton, P. W. Bateman

**Affiliations:** 1 School of Molecular and Life Sciences, Curtin University (Bentley), Perth, Western Australia 6102 Australia; 2 ARC Centre for Mine Site Restoration, School of Molecular and Life Sciences, Curtin University (Bentley), Perth, Western Australia 6102 Australia

## Abstract

Predator-prey interactions may be altered under human-induced rapid environmental change, such as urbanisation. Extensive clearing in urban areas may leave short-range endemic species, such as mygalomorph spiders, more vulnerable to local extinction through predation in remaining remnants. Predation rates on Australian mygalomorph spiders were assessed using clay models of two size classes (5 cm, 3 cm), during two time periods in 2016 (January–February, July–August). Size and phenology of models resembled the mygalomorph genera *Aname* and *Teyl* occurring in these local urban remnants. Local predator guilds were significantly influenced by leaf-litter cover (%) and proportion of surrounding parkland. Preference for spider vs. control models was consistent across all predator types (bird, rodent, lizard and wasp), but specialist spider wasps (Pompilidae) only attacked spider models. Generalist predators (birds, lizards and rodents) were more opportunistic. Lizards and rodents exhibit similar predation behaviour, indicating there may be some inter-specific competition. Invasive generalists (e. g. rodents) or urban adapters (e. g. corvids) are more likely to represent an increased threat to spiders than are co-evolved specialists (e.g. spider wasps).

## Introduction

Since 1960 the global human population has dramatically increased and consolidated in urban centres^1,2^, contributing to new processes that may threaten fauna^3,4^. Human-induced rapid environmental change, such as urban development, can place severe selective pressures on species to adapt to these changes, move away or persist in fragments or refugia within altered landscapes^5,6^.

Response to urban environments varies amongst taxa. Although presence of many species is positively correlated with distance from urban environments^7^, some taxa, known as ‘urban exploiters’ e.g. rock pigeons (*Columbia livia*) and some rodents (e.g. *Mus musculus* and *Rattus* spp.) are found mainly in urban landscapes, where they subsist on anthropogenic resources. Other species, e.g. red foxes (*Vulpes vulpes*) globally^8^ or ravens (*Corvus coronoides*) and butcherbirds (*Cracticus* spp.)^9^ in Australia can be termed ‘urban adapters’ and benefit from resources such as anthropogenic food or shelter in urban areas but are not limited to urbanised areas. Urban adapters may have profound impact on their native prey species that still persist in patches of urban bushland. While urban adapters may thrive in various landscapes surrounding urban bushland, many native species are restricted to such patches. Negative impacts on native taxa may come from either novel threats (invasive species) or changes in predation behaviour of other native taxa through urbanisation.

Taxa with comparatively low mobility, low-fecundity, poor dispersal and small geographic range may persist in very small natural habitat remnants in urban areas if the quality of the patch is maintained^10^, and may be referred to as ‘urban engulfed’. These traits are present in many species of millipedes, snails, cicadas and mygalomorph spiders^11^. Such species are known as short-range endemics (SRE)^12^ and are of high conservation priority in Australia^13,14^.

SRE species may face extinction as a result of the additional pressure of predation in small, fragmented populations. Differential predation of taxa across urban areas has not been well documented. Research addressing the threat associated with invasive rodents in Australia has often focused on various impacts on native taxa^15–18^. There is evidence that invasive rodents can have a severe effect on invertebrates on islands^19^, which may be analogous to predation in urban bushland fragments, as many native species may be confined to a single fragment. Reptiles, and lizards in particular, are predators of spiders and other invertebrates^20^. Reptile response in an urban environment seems to vary markedly between species, with some skinks being urban exploiters or adapters^21^. In Australia, predator-prey interactions involving invertebrates is less studied than vertebrates, probably due to taxonomic impediment and bias towards more charismatic subject species^14,22^. However, the predation and parasitisation of spiders by wasps (typically members of the Pompilidae), has been long known^23^. Exploring predator-prey interactions between urban wildlife will assist in understanding complex interactions, and how these may vary under HIREC.

**Figure 1:**
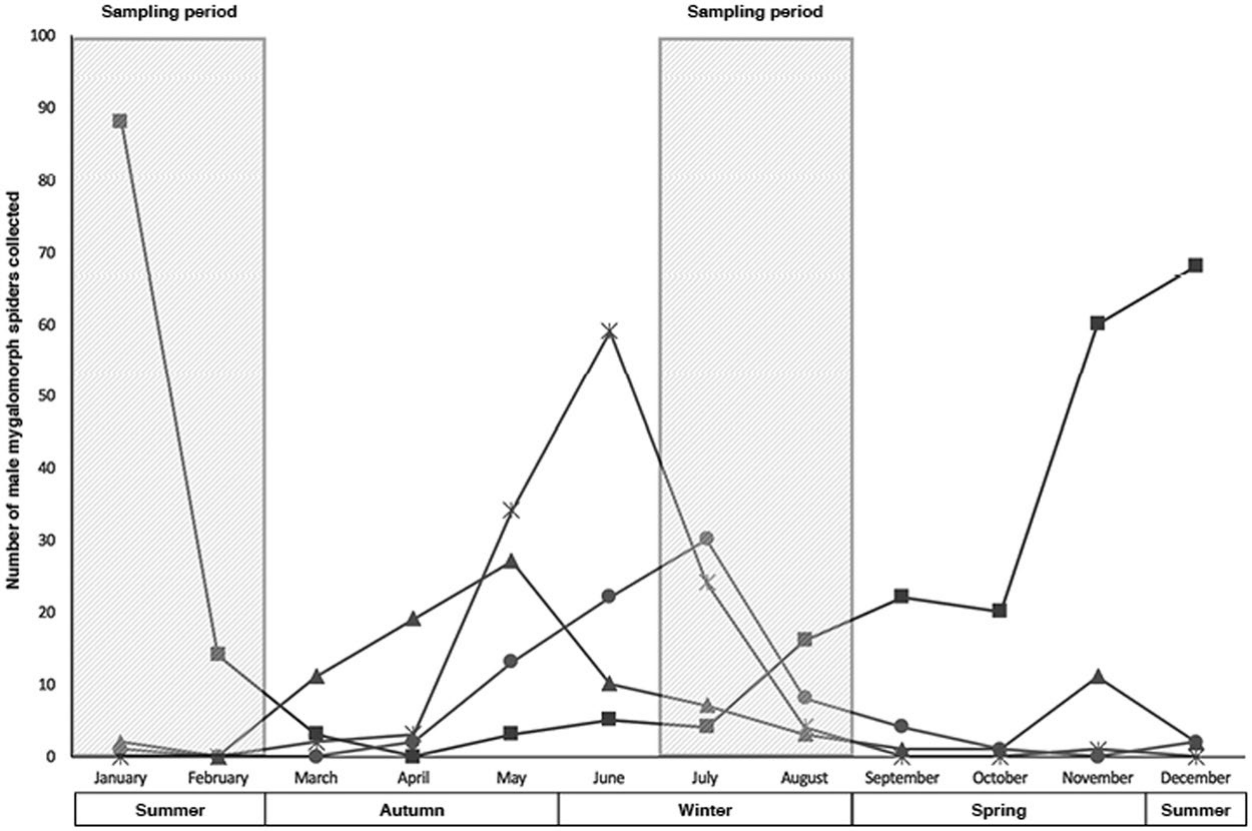
Phenology of males of four mygalomorph families (Actinopodidae [×], Barychelidae [▲], Idiopidae [●] and Nemesiidae [■]), collected in Perth, south-western Australia while roaming. Records used from Atlas of Living Australia (ALA) for all years.

We sought to explore variation of predation on quintessential SRE taxa - mygalomorph spiders - across urban remnant bushland patches. Male mygalomorph spiders reach sexual maturity in 5–7 years after hatching, depending on species^24^. Subsequently, adult males emerge and roam to mate. This venture is a highly seasonal occurrence and appears to vary between species (Fig. 1).

For most species in Perth, south-western Australia, male roaming occurs during periods of high humidity in winter, possibly due to physiological constraints^25^. However, one species (Nemesiidae: *Aname mainae)*, roams during the summer months. *A*. *mainae* is the largest mygalomorph species of the region, reaching up to 5 cm in length. Males of most other species measure up to 2–3 cm in total body length^24^. These male dispersal events are likely to be the most dangerous times for mygalomorphs as they are exposed to a suite of predators to which they would not be exposed when in burrows. We aimed to test if there were selective or increased seasonal predation of male mygalomorphs while they are exposed on the surface during the mating season. Mygalomorph spider species have specialised microhabitats requirements. Hence, factors such as leaf litter, mid-storey and canopy cover may vary between species in their exposure to predators. In conjunction with other increased pressures present in an urban context, predation may decrease the chances of ongoing persistence of SREs.

We used clay models to explore the effects of habitat patch size, microhabitat and seasonal predation on spiders in urban areas. Clay models have been successfully used to assess predation on small taxa such as lizards, snakes, mice, invertebrates and bird eggs^26,27^. As SRE endemic taxa are of high conservation priority and predation marks can be confidently identified on clay models, we decided this was the most effective and least harmful approach to test our hypotheses^27^. Our study is the first to use clay models to measure predation types, predation size and frequency on mygalomorph spiders. The questions we asked were:

1. What are the local predator guilds?

- Is there evidence of competition between identified predator types?
- Is surrounding land-use correlated with guilds?

(2) Is there a significant difference between predator preferences in terms of spider vs. control, size of model, and season?
(3) Is predation frequency by identified predator types influenced by microhabitat variables such as leaf litter, mid-storey and canopy cover?
(4) Does identified predator type vary in terms of number of attacks or attack location on the body of the spider?

## Results

Of the 2400 models used, 663 (28%) were attacked. From marks on the models predators were identified^28^ as birds (33%), lizards (25%), rodents (29%) and wasps (13%).

### Local predator guilds

**Figure 2:**
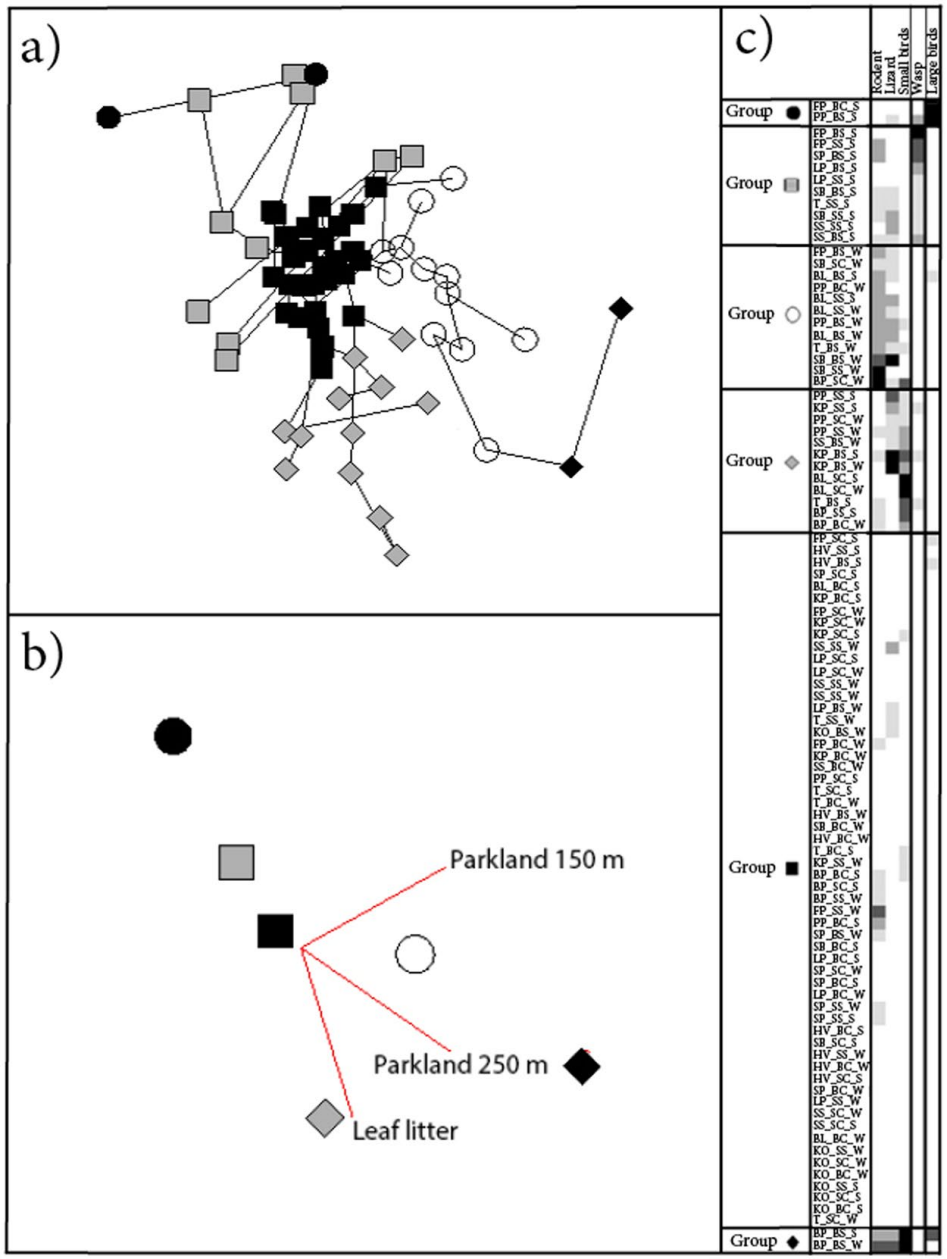
2D semi-strong hybrid (SSH) multidimensional scaling (MDS) ordination (Stress: 0.18) based on ninety-six ‘patch-model-season’ units and five predation types (Gower Metric Association Measure, Classification Strategy: Agglomerative Hierarchical Fusion, Technique: Flexible UPGMA). Predation types were used as intrinsic factors and combined patch, model type and season were used to separate units. Each “site_model_season” unit presents twenty-five models in the two-way table and are coded as follows: the first line refers to the patch of urban bushland and corresponds to the site map (Fig. 4), the second line of codes refers to model type (BS: 5 mm spider, BC: 5 mm control, SS: 3 mm spider and SC: 3 mm control). The third line of code refers to season (S: Summer or W: Winter). (**a**) Significant (MCAO, P < 0.001) extrinsic factors (leaf litter and proportion of surrounding parklands - buffer 150 and 250 m around patches) were fitted using principal PCC. (**b**) Six distinct groups emerged based on predation intrinsic factors. (**c**) Two-way table.

Local predator guilds were defined based on similar predation behaviour across ninety-six model-patch-season units. Six distinct groups were recognised (Fig. 2): group 1 included two ‘model-patch-seasons’ units and was based on ‘large birds’; group 2 included 10 model-patch-seasons and clustered based on ‘wasp’ predation. Similarly ‘lizards and rodents’ predation formed group 3, and consisted of twelve spider-patch-seasons units. Group 4 included twelve model-patch-seasons and was based on predation by ‘lizards and small birds’. Group 5 included fifty-eight model-patch-seasons and was formed through absence of predation. Group 6 included two model-patch-seasons and was formed from high predation of large spider models across ‘all identified predator types, except wasps’, in Bold Park in both summer and winter.

Three extrinsic factors fitted using principal component correlation (PCC) were significant using Monte-Carlo attributes in an ordination (MCAO): leaf litter cover and proportion of parkland surrounding the patch within a 150 m and 250 m zone (Fig. 2).

### Predator predilections

Predator type was modelled using a multinomial logistic regression (MLR). Overall model accuracy were moderate (accuracy 0.70, kappa 0.58, df 72) with significant variables reported in Table 1.

**Table 1:** Model type preference (control or spider), size (large or small) and season (winter or summer), and frequency of predation by identified predator type (bird, lizard, rodent or wasp).

	Preference		Size		Season	
	Control	Spider	Large	Small	Summer	Winter
Bird	88	128	121	95	84	130
Lizard	34	132	96	70	71	95
Rodent	49	143	113	79	82	110
Wasp	0	89***	55	34	81****	8

Multinomial logistic regression was used to determine significance ***p < 0.001, and ****p < 0.0001.

Wasp attacks were found only on spider models (b). Wasps were the only predator that showed significant (p < 0.0001) seasonal differences, predating significantly more in summer than in winter (Table 1).

### Microhabitat variables

Percentage cover of microhabitat variables significantly influenced the predation of models by predator types. Wasps predated spider models significantly more in areas with lower canopy cover (Table 2). Rodents predated spider models significantly more in low percentage understorey canopy cover (Table 2). Lizards predated spider models significantly more in areas with high leaf litter and canopy cover (Table 2). All birds predated spider models significantly more in low leaf litter and in lower understorey cover areas (Table 2).

**Table 2:** Summary of predation type in different microhabitat strata cover (%: leaf litter, understorey and canopy). Values are presented as Mean ± Standard deviation.

	Leaf litter	Understorey	Canopy
Bird	72.7 ± 30.6*	31.8 ± 20.6**	37.7 ± 26
Lizard	77.3 ± 29.7*	32.7 ± 21.8	40.2 ± 27.9**
Rodent	70.5 ± 34.3	28.7 ± 23.2*	34.7 ± 28.2
Wasp	72.8 ± 31.7	38.4 ± 23.5	22.6 ± 25.4****

Multinomial logistic regression was used to determine significance, *p < 0.05, **p < 0.01 and ****p < 0.0001.

### Predator attack

The majority of models were attacked more (attack number) than once by a single identified predator type. However, attack number varied significantly among identified predator types (Table 3). Birds, lizards and rodents typically attacked a single model between 1–4 times. Conversely, wasps typically attacked models over 8 times - significantly more than attack number categories (Table 3).

**Table 3:** Location of attacks on models for each identified predation type.

	Attack location		Attack number		
	Edge	Middle	1–4	5–8	>8
Bird	61	82	170***	16	4
Lizard	183***	46	135***	12	3
Rodent	192***	59	8	2	66***
Wasp	2	89***	170***	16	4

Numbers reflect the total number of attacks for each identified predator type within each category. ***p < 0.001.

Bird attack rates were similar for both middle and edges of models (Table 3). Wasps attacked the middle significantly more than they did the edge of the models (Table 3). Lizards and rodents, however, attacked edges significantly more than the middle of models (Table 3).

## Discussion

A clear preference for non spider-mimicking controls by all identified predator types indicated that predators were selecting prey based on visual cues, and not only due to curiosity towards foreign objects. The use of controls is surprisingly rare in experiments involving clay models, but is encouraged^29^.

### Local predator guilds

Predator guilds can have a great impact on prey species^30^ and such guilds may also interact with each other, influencing their impact on prey^31^. Prey species that are vulnerable to disturbance^10^ may be further impacted by changes in local predator guilds. Disturbance can also change local predator guilds by disrupting landscape structure and resources that were previously stable^31,32^. Generalist predators may be less affected by disturbance than specialists as their broad prey spectrum does not restrict their activities in any particular area^33^. Amount of parkland surrounding patches significantly influence predator guilds, increasing towards guilds of predominantly rodents and lizards (Fig. 2: Group O). This may indicate use of surrounding areas such as parklands by these predators, possibly as territory extension outside the patch or to avoid roads and buildings.

### Predator predilections

In addition to rodent, bird and lizard bite marks - as has previously been recorded in multiple studies using clay models^29^ - we also found small piercings on many models attributed to spider-hunting wasps. Wasps were only predator with a significantly higher predation rate in summer. Wasps visually recognised their prey^34^ as attacks were restricted to spider models; no control models were attacked. Smaller models may be less detectable or may have been preyed upon by different wasp species, indicating a suite of spider-hunting wasp species.

Pompilidae, the family of wasps that prey on spiders, is thought to be highly specific to the species or size of spider prey^35^, perhaps due to constraints in wasp and/or nest size^35^. The pompilid *Cryptocheilus bicolor* is commonly seen throughout Australia dragging huntsmen spiders (Sparrasidae) back to its lair. Records of pompilids collected in Perth (ALA, n = 20) correlate with *Aname* males roaming phenology (October to February), though they also occur through to April. Wasps may not be targeting specific spider species, but a specific size of prey which may depend on size of the wasp^35^. For example, *C*. *bicolor* may have been attacking large models mistaking them for sparrassids of a similar size, rather than targeting mygalomorphs. Both *Aname* and some sparrassids, such as *Dingosa*^36^, have open burrows for which wasps may display similar searching behaviour. However, spider response varies – *Dingosa* avoid wasps by running out of their burrow (pers. obs.), whereas mygalomorphs are more likely to defend burrows through phragmosis i.e. defend the burrow using their body. Predation rates on models are limited in this way as they do not reflect actual outcomes of encounters between wasps and prey.

### Microhabitat variables

Microhabitat variables and location of predation events indicate that while rodents and lizards predate at similar rates in the same patches, they occupy different microhabitats. Lizards preferred to forage in areas with high leaf litter and canopy cover, while rodents preferred to forage primarily under high understory cover. Location of lizard and rodent bite marks on the edge of control models or on the legs of model spiders probably reflects the approach of these predators at ground-level. Most lizards are active during the day, but rodents are nocturnal. As mygalomorph spiders are thought to roam primarily at night (except for *Missulena* which has bright red jaws), predation by rodents may be relatively common. Although bats are known to predate on spiders^37^, their contribution to predation rates is not known and no bat bite impressions were identified in this study. Predators may use senses other than visual cues to locate their prey^38,39^. We suggest future predation experiments either test these factors or be mindful when interpreting results.

### Predator attack

Bird predation varied between large birds and small birds in local predator guilds (Fig. 2), but not foraging behaviour (Table 1). Large birds (>2 mm peck marks), perhaps due to high mobility did not have clear associations with other identified predator types (Fig. 2). Bird attack rates suggested significantly higher predation in microhabitats with low leaf litter and understorey cover which may reflect a foraging strategy from an above ground vantage point. Similarly, location of attacks on models was seemingly lacking discrimination when compared to ground-dwelling predators.

### Implications

As a novel threat, predation by invasive rodents is of high conservation concern^15,18,19^ and would affect all local trapdoor spider species, regardless of season. Displacement and local extinction of native nocturnal predators (multiple small and medium-sized dasyurid carnivores) since Perth was established makes it impossible to know if predation rates are higher, similar or lower than before urbanisation. It is possible that rodents living in native bushland remnants have a similar deleterious effect as they do on islands^19^. The adaptation of natural predators such as ravens and magpies in urban areas^40,41^ may also have a profound impact on spider populations, but potentially only on spiders active during the day (*Missulena*). High predator mobility, in conjunction with a higher abundance, may be especially detrimental to species restricted to smaller patches^12,14^. We found no significant difference in predation type or rate with size of patch, which is worrying, as it suggests that populations in smaller patches may have a higher extinction debt^42,43^.

It is possible that predation rates by different taxa are not indicative of an individual attacking one model. For example, a raven may learn quickly that models are not suitable food sources after the first instance. Conversely, a wasp may not learn and continue to attack multiple models in the same area indefinitely. As wasps are highly mobile, attacks on multiple spider models over one quadrat may actually indicate low levels of learning, rather than a high density of wasps. Similarly, a slow-moving lizard, such as a bobtail (*Tiliqua rugosa*), may encounter only a few models during the week, whereas a fast-moving territorial lizard, such as a scrubland skink (*Morethia obscura*), may encounter many models and multiple times. Site characteristics such as size and microhabitat variables may be accurate indicators for adequate territory ranges for different species. For example, rodents may require a much lower territory range to satisfy their diets than would corvids because of the vast differences in size and mobility capabilities. For future studies, it is advisable that predator range, if known, be taken into account when interpreting results in regards to size and connectivity of patches.

As pompilid wasps are native, and have co-evolved with native spider species it is unlikely that the impact of their predation on spiders is unsustainable. Indeed, this may be a positive finding for wasp conservation in that, as natural predators of spiders, wasps are still maintaining adequate numbers for survival in an urban context. Similarly, if parasitic wasps are maintaining adequate numbers then abundance of spider prey species are, at present, likely to be sustainable in order to support wasp populations.

### Conclusions

Birds, lizards and rodents are all generalists and operate on different scales making it difficult to assess their realistic impact on spiders. Pompilid wasps are specialists and, although highly mobile, operate on a similar size scale to the spiders they prey on. However, as wasps have co-evolved with spiders, predation from wasps will be limited by spider populations. Invasive generalist species such as rodents or urban adapters such as corvids are more likely to be a new threat to spiders than co-evolved specialists. It is recommended that replication of this study is conducted over time and in areas with no invasive species to determine changes in rates or proportion of predation. We conclude that the impact of predation on spiders may not in itself be a threat to their population persistence, but may exacerbate existing threats such as habitat loss, invasive grass and impacts of fires.

## Methods

### Study sites

**Figure 3:**
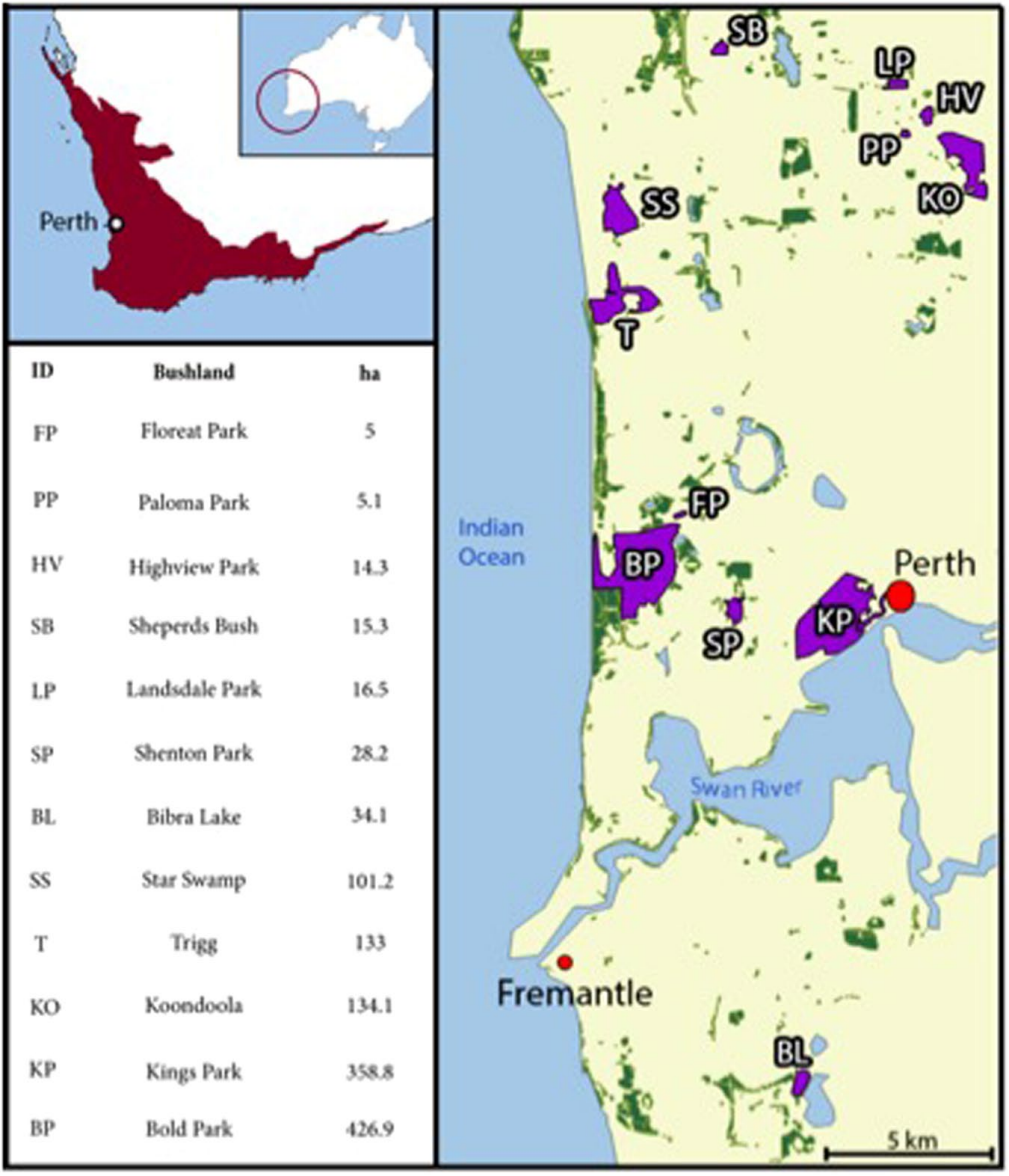
Twelve urban remnant bushland sites in Perth urban area used to test predation on clay models. Perth is situated within the biodiversity hotspot of South-Western Australia (SWA).

All study sites were located in Perth, a highly urbanised centre with a Mediterranean climate (mean annual rainfall 740 mm), located in the South-west Australian (SWA) Global Biodiversity hotspot^44^. Twelve urban remnant sites were selected to provide a range of size categories (Fig. 3).

To test any effect of season, sampling was conducted during two different time periods in 2016 (January–February, and July–August). Size and phenology replicate real mygalomorph species that occur in the urban remnant vegetation of Perth (Fig. 1).

### Plasticine models

**Figure 4:**
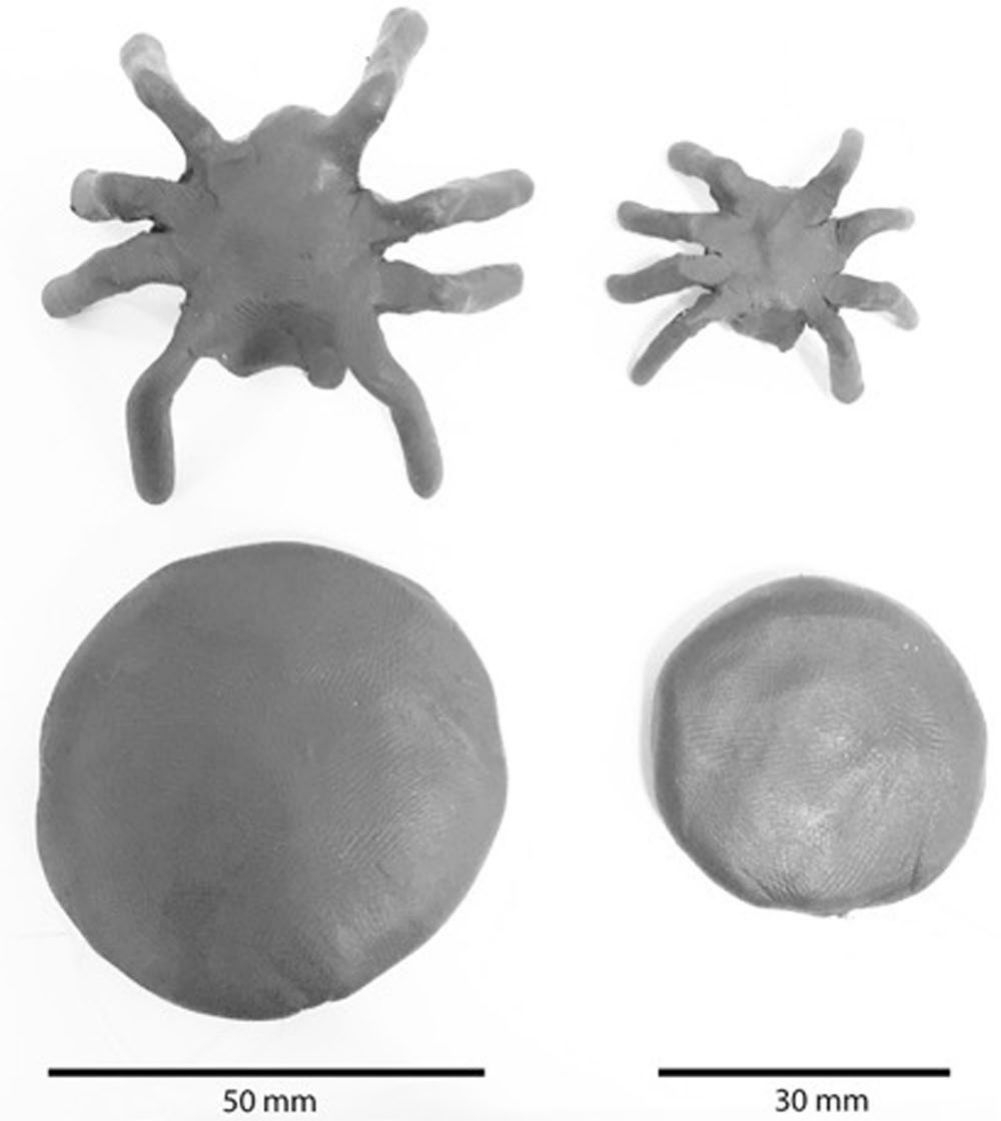
3D printed spider models and washers of two size classes covered in black plasticine clay.

Predation rates were assessed using 2400 clay models of two size classes (3 cm and 5 cm) (Fig. 4) and types (spider and control) during two sampling periods. To standardise, spider models were printed with plastic resin using a 3D printer using TinkerCad and UP! Software. This 3D model is available online through TinkerCad as “Spider Model by Leanda Mason”. Metal washers of similar size classes were used as controls. Models and washers were uniformly coated with a layer of black plasticine (Flair Leisure Products). Models were connected by transparent fishing wire to a nail pushed into the earth, securing both the model’s location and a numbered tag.

**Figure 5:**
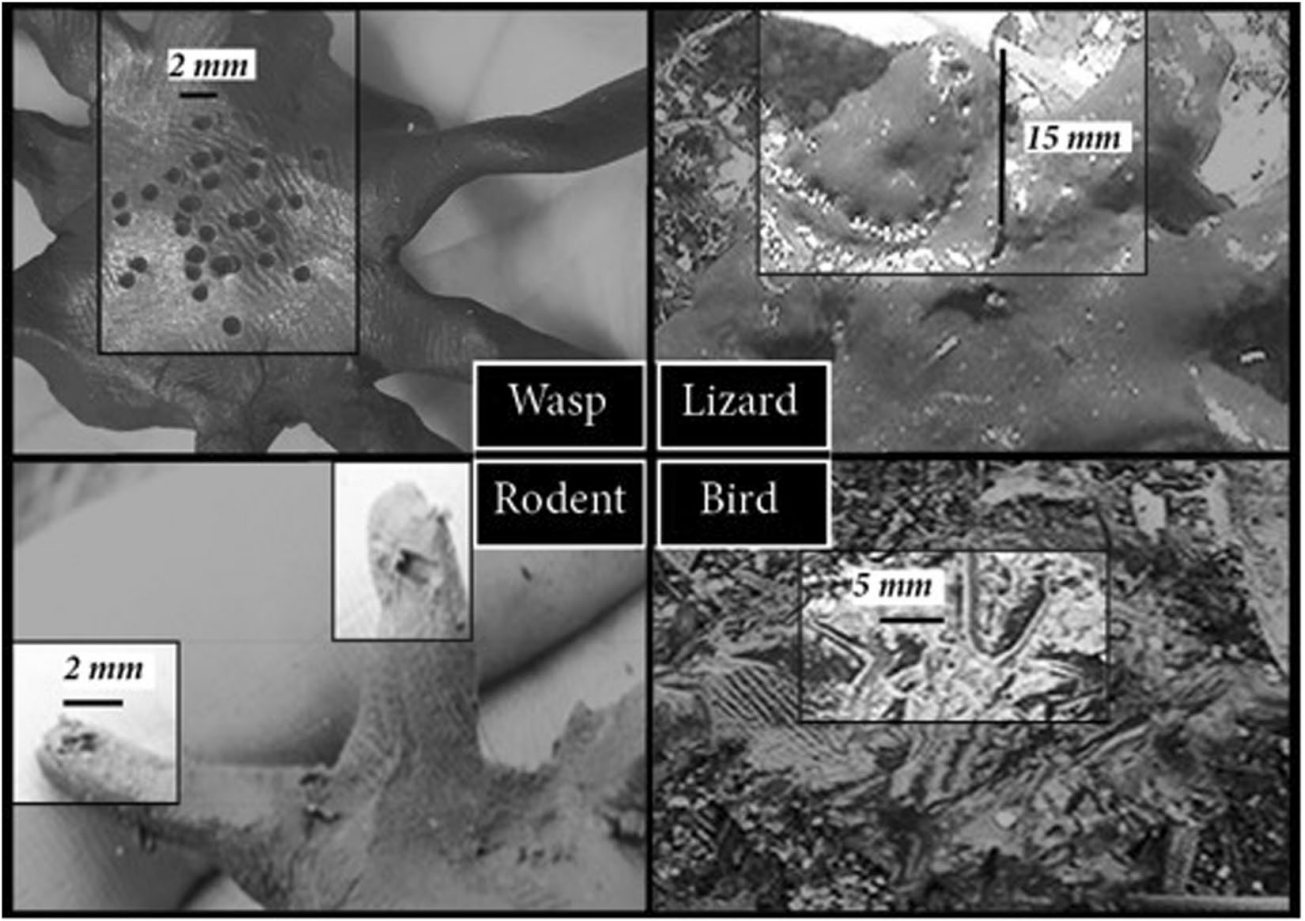
Impressions left by predators on clay models in urban remnants in Perth, south-western Australia were identified, measured and location recorded.

Twenty-five of each of four treatments; small spider, large spider, small control, large control, were placed in a 100 m × 100 m quadrat 10 m apart. Predation type (bird, lizard, rodent and wasp), size (mm), location on the model and number of attacks were identified from distinctive marks left by predators on the clay models (see Fig. 5).

### Microhabitat and patch variables

Leaf litter, mid-storey vegetation and canopy vegetation were recorded. We assumed this was related to the visibility of models to predators. Surrounding land-use area was measured by generating shapefile layers of buildings, roads, parkland and other remnant vegetation within 250 m, 150 m and 50 m buffers. Buffers were measured for both patch and quadrats in the open source program Quantum Geographic Information System (QGIS 2014). The Nearmap plugin was used to determine the proportion of different land-use within each buffer area.

### Statistical analysis

As this study examines predator behaviour we used predation as intrinsic factors, and determined whether site variables correlated with the resulting multidimensional scaling ordination axes in numerical taxonomic analysis^45^, using PATN3.11^46^. All 2400 models were included in the cluster analysis (predated and non-predated) and converted into a proportion within each unit. Predator types were used as intrinsic factors and combined patch, model type and season were used as separate units (25 models in each unit). The Gower metric was used to determine the degree of similarity between different identified predator types, followed by hierarchical polythetic agglomerative clustering using flexible UPGMA. We used the Two-Step association measure to determine influence of variable groups on identified predator types. A two-way table is a visual representation of the association between different units (guilds) and the influence of identified predator types to form groups. Darker cells show greater association than lighter cells. MDS ordination portrays the spatial relationship between objects in a way that best preserves relative positions. ‘Stress’ measures the level of distortion from reducing axes (to two in this case), with low stress values (i.e. <0.20) indicating better representation of positions than high stress. The Minimal Spanning Tree (MST) is a form of network analysis that connects each object to its nearest neighbours. If there is a high congruence between cluster analysis, ordination and network analysis, then resulting categories are supported. This allows ‘local guilds’^32^ of predators to be determined and analysis of the influence site variables. Competition within local predator guilds can then also be assessed. During analysis, we found that attack marks from bird species on the clay measuring ≤2 mm and >2 mm birds diverged. Extrinsic factors were fitted to the ordination using PCC and significance determined using MCAO. The strong clustering formed by using patch, model type and season as units indicated that these factors may be useful in predicting identified predator type behaviour through multinomial logistic regression.

A MLR was fitted (package nnet, R 3.3.0) using predated data only (n = 510). MLR require a nominal dependant variable with more than two levels and can handle categorical variables^47^. Model coefficients (logit) represent the change in log-odds of a variables influence relative to the reference category (in this case “birds”). A positive logit indicates the effect of the predictor on predation (relative to birds) is positive. We have used “b” to represent the logit in results. Predator type (bird [145], lizard [139], rodent [150], wasp [76]) was the response and site, site size, season, model size and type, attack number, attack location and litter, understory and canopy cover the predictors. Data was split into a train and test set (80% train, 20% test) and the packages ‘caret’ and ‘e1071’ used to assess classification accuracy. The package stargazer^48^ was used to plot significance levels. Predator size categories were reduced for analysis to: 1 (A), 2 (B) and >2 (C). The location of predation was standardised between model type: ‘Body’ of spider models were subsumed into ‘middle’, and spider model ‘legs’ into ‘edge’. The number of attacks was categorised into: 1–4 (A), 5–8 (B) and >8 (C). A multinomial logistic regression was preferred over other GLM’s as several predictors were categorical and the assumptions of normality and homoscedasticity could not be met. Additionally, as model size and type were recorded at the within plot scale and were to be included in the analysis, data was not averaged to predation rate per plot.

## Data Availability

All data can be made available to access upon publication.
